# Microvascular Free Flap Transfer for the Closure of Recalcitrant Digestive Fistulas: A Systematic Review of Surgical Success, Definitive Closure, and Clinical Outcomes

**DOI:** 10.7759/cureus.114687

**Published:** 2026-08-17

**Authors:** Alexis Quetzalcoatl Vega Morales, Zeus Edrian Daniel Alfonso González Mercado, Benjamin Jared Franco Bautista, Jefferson David Gutierrez Parrado, Milton Fernando Crespo Macias, Cecilia Lucia Rojas Fabara, Ana Laura Esmeralda Munoz Avendano, Valeria Garzón Burgos, Paola Andrea Quispe Yepes

**Affiliations:** 1 General Surgery, University of Guadalajara, Mexican Social Security Institute (IMSS) Regional General Hospital No. 180, Guadalajara, MEX; 2 Surgery, Instituto Mexicano del Seguro Social, Ciudad de Reynosa, MEX; 3 Medicine, Universidad Regional del Sureste, Oaxaca, MEX; 4 Medicine, Universidad de Santander, Cúcuta, COL; 5 General Medicine, Centro de Salud Familiar (CESFAM) Batuco, Santiago de Chile, CHL; 6 Medicine, Ministry of Public Health, Quito, ECU; 7 Surgery, Hospital de Especialidades Puebla, Puebla, MEX; 8 Medicine, Unisanitas, Bogotá, COL; 9 Medicine, Universidad del Rosario, Bogotá, COL

**Keywords:** digestive fistula, enterocutaneous fistula, free microvascular flap, free tissue transfer, pharyngocutaneous fistula, plastic and reconstructive surgery, systematic review

## Abstract

Recalcitrant digestive fistulas are difficult to manage when local tissues are irradiated, infected, scarred, or depleted. Because pharyngocutaneous, enterocutaneous, esophageal, colorectal/perineal, and pancreatic fistulas are anatomically and biologically distinct, they were evaluated as site-stratified evidence rather than as a single pooled clinical entity. This review aimed to assess definitive fistula closure, flap survival, recurrence, complications, reoperation, mortality, and functional recovery after microvascular free flap transfer for established recalcitrant digestive fistulas. This Preferred Reporting Items for Systematic Reviews and Meta-Analyses 2020-informed qualitative systematic review searched PubMed/MEDLINE, Embase, Scopus, Web of Science Core Collection, the Cochrane Library, Google Scholar, and reference lists. Heterogeneity in fistula anatomy, timing, flap selection, adjunctive procedures, and outcome definitions precluded a pooled meta-analysis. The review included 15 studies, including seven case series, six case reports, one retrospective cohort, and one technical clinical report, with sample sizes ranging from 1 to 50. Direct evidence was concentrated in post-laryngectomy pharyngocutaneous or pharyngostome fistulas. In studies with explicit extractable numerators, 25/25 flaps survived, but this figure derived from three small series and six case reports and excludes larger cohorts with incomplete or nonseparable reporting. Larger series reported substantial complications, revisions, and adjunctive procedures. Abdominal enterocutaneous evidence was limited to isolated salvage cases. Microvascular free flap transfer is a plausible salvage option for selected recalcitrant digestive fistulas, particularly in irradiated or surgically depleted head and neck fields. The evidence remains very low in certainty and does not support a single pooled closure estimate across fistula sites.

## Introduction and background

Digestive fistulas are abnormal epithelialized communications between the gastrointestinal tract and the skin or an adjacent organ and are among the most morbid complications in reconstructive and gastrointestinal surgery. Although anorectal fistulas have dedicated site-specific guidelines [1], the present review concerns recalcitrant alimentary-cutaneous fistulas requiring microvascular reconstruction; these anatomically distinct entities include enterocutaneous, pharyngocutaneous, esophagocutaneous, colorectal, and pancreatic fistulas and present different physiologic and reconstructive challenges. A systematic review reported pooled mortality of 14.1% (95% confidence interval: 11.2-17.5) and spontaneous closure in 34.6% of cases despite multimodal therapy [2]. A prospective 25-patient cohort reported closure in 21/25 (84%) patients and mortality in 4/25 (16%) patients [3]. These findings illustrate the treatment gap in complicated and chronic fistula disease.

For this review, a recalcitrant fistula was operationally defined as an established fistula persisting or recurring after standard medical, nutritional, endoscopic, local, or regional surgical management, generally for at least three months when duration was reported. The burden extends beyond the anatomic defect. In a 62-patient quality-of-life study, the 36-Item Short Form Health Survey domain scores were lower than those of matched controls [4]. A national readmissions analysis reported median acute-care hospital charges of $55,469.83 in patients with enterocutaneous fistula versus $19,625.06 without fistula, corresponding to an attributable inpatient burden of $35,844.77 per patient [5]. This overlap between clinical failure, dysfunction, and resource use has increased interest in biologically robust reconstruction, including microvascular free flap transfer.

Microvascular free flaps provide vascularized tissue that can obliterate dead space, reinforce suture lines, replace irradiated or fibrotic tissue, and introduce viable tissue into chronically infected or poorly perfused fields [6]. Site-specific reports have used anterolateral thigh, radial forearm, rectus abdominis, jejunal, latissimus dorsi, and composite flap designs to replace lining, external cover, or both. Prior reviews have reported favorable outcomes in selected enterocutaneous, pharyngocutaneous, esophageal, colorectal/perineal, and pancreatic settings, but their eligibility criteria and anatomic coverage differ substantially [7-11].

The evidence remains fragmented. Etiology, microbial load, tissue vascularity, prior irradiation, nutritional status, recipient-vessel availability, reconstructive objectives, and functional endpoints differ across fistula sites. Accordingly, this review did not assume a universal closure rate and instead compared the strength of evidence by fistula location and reconstructive indication. The primary objective was to assess definitive closure after microvascular free flap transfer for recalcitrant digestive fistulas. Secondary outcomes were flap survival, recurrence, complications, reoperation, mortality, functional recovery, and risk-of-bias patterns.

## Review

Methodology

Study Design and Reporting Framework

This systematic review assessed the clinical success of microvascular free flap transfer for established recalcitrant digestive fistulas and was developed using the Preferred Reporting Items for Systematic Reviews and Meta-Analyses (PRISMA) 2020 reporting framework [12]. Because the evidence consisted predominantly of retrospective series, small case series, and case reports, qualitative synthesis was prespecified. Quantitative pooling was considered only when studies reported comparable numerators, denominators, outcome definitions, fistula sites, and follow-up periods.

No prospective protocol registration record was available in the submitted source material. The documented search included literature available through July 2026. An exact day-level search log was not retained, and this is acknowledged as a reproducibility limitation.

Eligibility Criteria

Eligible studies were human clinical reports involving pharyngocutaneous, pharyngostome, oropharyngocutaneous, esophagocutaneous, enterocutaneous, or related alimentary-cutaneous fistulas described as persistent, refractory, recurrent, complex, salvage, post-radiotherapy, post-oncologic, post-surgical, or otherwise recalcitrant. Studies had to use microvascular free tissue transfer to treat an established fistula and report at least one clinical outcome, including definitive closure, flap survival, recurrence, complications, reoperation, mortality, or functional recovery.

Animal and cadaveric studies, narrative reviews, editorials, letters without patient-level outcomes, prophylactic primary reconstruction, local or pedicled flap treatment without separable free-flap outcomes, negative-pressure therapy alone, and conservative treatment without free-flap reconstruction were excluded.

Information Sources and Search Strategy

PubMed/MEDLINE, Embase, Scopus, Web of Science Core Collection, the Cochrane Library, and Google Scholar were searched. Reference lists of eligible studies and recent relevant reviews were also screened to identify older microsurgical reports not retrieved by keyword searching. The PubMed strategy was (“Free Tissue Flaps”[Mesh] OR free flap* OR pharyngostome OR pharyngoesophageal OR esophagocutaneous OR oropharyngocutaneous OR gastrocutaneous OR pancreaticocutaneous) AND (closure OR repair OR reconstruction OR salvage OR recalcitrant OR persistent OR recurrent). Database-specific subject headings and title/abstract/keyword fields were used for Embase, Scopus, and Web of Science. Google Scholar combinations included pharyngocutaneous fistula free flap, pharyngostome free flap, enterocutaneous fistula free flap, microvascular fistula closure, and free jejunal patch pharyngocutaneous fistula.

Conference abstracts identified through supplementary searching were screened, but grey literature was not searched systematically. The English-language restriction and the absence of a preserved database-by-database export introduced potential language and database-retrieval bias.

Study Selection and Data Extraction

Titles and abstracts were assessed against the eligibility criteria, potentially eligible full texts were retrieved, and reports were categorized as directly eligible or contextual. Directly eligible studies used free flap transfer to close or reconstruct an established digestive fistula. Mixed studies were retained for narrative interpretation when free flap-specific outcomes could not be completely separated, but they were not used for pooled estimates.

The source records did not document independent duplicate screening or duplicate data extraction, and no disagreement resolution procedure was available for verification. This limitation is now stated explicitly rather than inferred.

Extracted variables were first author, publication year, country, design, sample size, fistula type and etiology, previous radiotherapy or surgery, fistula duration when reported, previous failed interventions, flap type, recipient-vessel strategy when reported, salivary bypass or enteral feeding adjuncts, definitive closure, flap survival, recurrence, complications, reoperation, mortality, oral intake or swallowing recovery, and follow-up duration.

Outcome Measures

The primary outcome was definitive fistula closure, defined as durable closure of the alimentary-cutaneous communication without external leakage at the final reported follow-up. Secondary outcomes included flap survival, recurrence, time to closure, partial or total flap loss, infection, dehiscence, anastomotic leak, reoperation, mortality, restoration of oral nutrition, nutritional recovery, speech or swallowing outcomes, and hospital length of stay.

Risk of Bias and Certainty of Evidence

Case reports and case series were appraised using an adapted Murad et al. framework [13], focusing on patient selection, ascertainment of the fistula and intervention, adequacy of outcome reporting, follow-up, and selective reporting. Retrospective cohorts were judged using the same domains adapted to uncontrolled surgical evidence. Consecutive recruitment, explicit denominators, defined outcomes, and adequate follow-up were considered lower risk than isolated successful cases with incomplete follow-up.

A formal Grading of Recommendations, Assessment, Development and Evaluations (GRADE) table was not undertaken because the directly eligible evidence was almost entirely uncontrolled and heterogeneous. Overall certainty was judged very low because of serious risk of bias, inconsistency, imprecision, indirectness across anatomic sites, and likely publication bias.

Data Synthesis and Statistical Analysis

Substantial heterogeneity was anticipated in fistula location, etiology, radiation exposure, timing of repair, flap selection, adjunctive procedures, and outcome definitions. Results were summarized descriptively and tabulated by fistula and flap type. Inferential tests and random-effects meta-analysis were not performed because comparable denominators, consistent outcome definitions, and sufficiently homogeneous subgroups were unavailable. Descriptive counts and percentages were used to characterize the evidence base, not to compare treatments or test hypotheses. No dedicated statistical software was used.

Even within the post-laryngectomy subgroup, definitive closure could not be pooled because larger studies variably combined flap survival, staged closure, adjunctive procedures, and functional recovery, and did not provide consistent free flap-specific denominators.

Results

**Figure 1:**
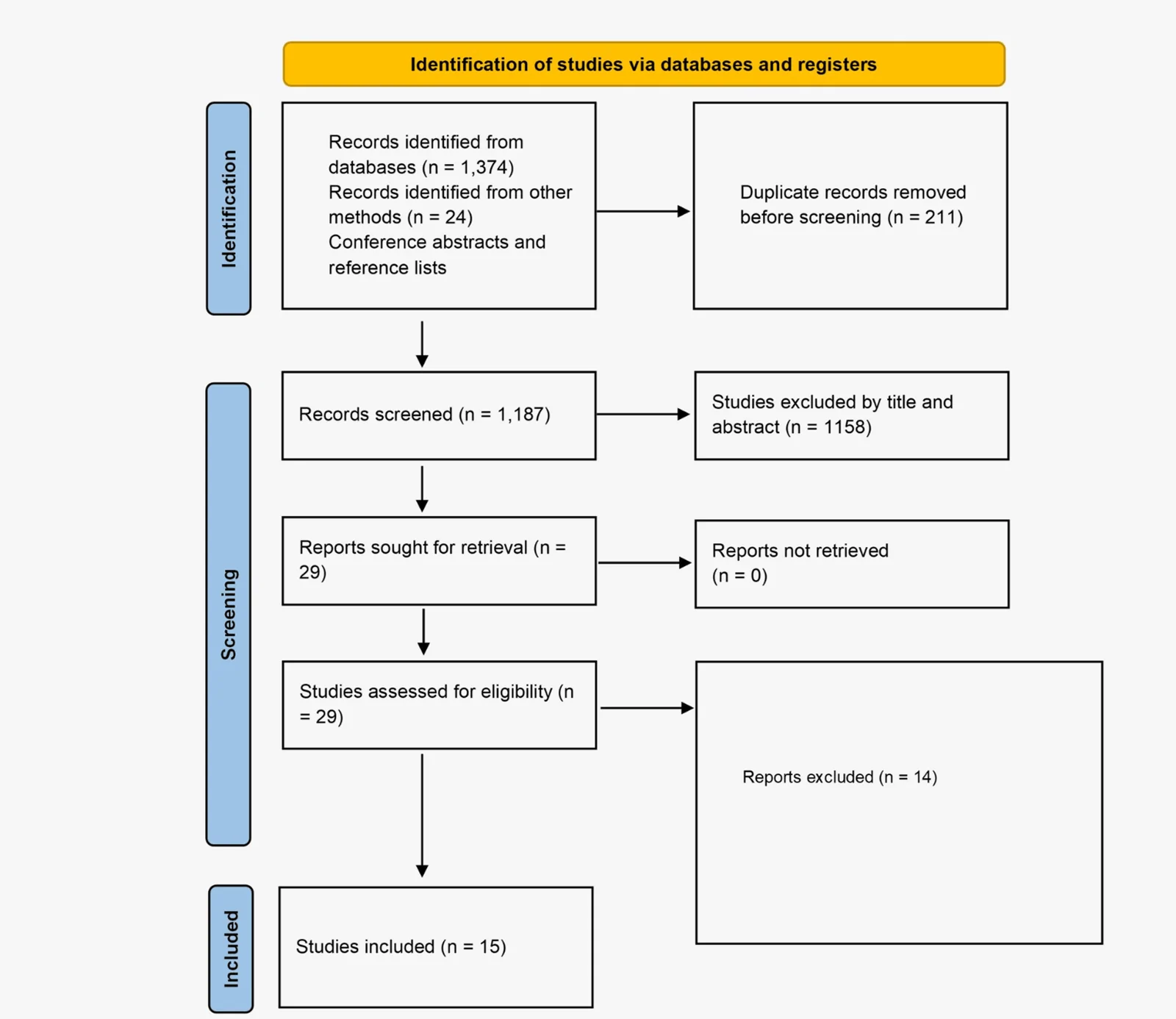
Preferred Reporting Items for Systematic Reviews and Meta-Analyses (PRISMA) flowchart illustrating study selection.

The evidence comprised retrospective case series, small case series, case reports, one technical report, and one mixed retrospective cohort. Head and neck reports used anterolateral thigh, lateral arm, radial forearm, dorsalis pedis, jejunal patch, and tissue-plug approaches [12-28]. A mixed post-laryngectomy cohort was retained for context because free flap-specific outcomes were only partly separable [24]. Abdominal enterocutaneous evidence was limited to complex salvage reconstruction [25-28]. Llorente et al. [14] reported the largest directly related pharyngostome series. Bohannon et al. [15] described a 20-patient post-laryngectomy pharyngocutaneous fistula series requiring free flap repair. Figure 1 presents the PRISMA flowchart of study selection.

Across the 15 studies, sample sizes ranged from 1 to 50. Patient demographics, fistula duration, prior repairs, radiation history, recipient-vessel strategy, and follow-up were inconsistently reported. These variables are therefore described when available rather than presented as complete comparative columns. Study characteristics are summarized in Table 1.

**Table 1:** Characteristics of included and directly relevant studies.

Author(s)	Year	Country	Sample size	Design	Key findings
Llorente et al. [14]	2020	Spain	50	Retrospective case series	Large pharyngostomes reconstructed with free flaps; oral feeding restored in most evaluable survivors, with frequent complications and revisions
Bohannon et al. [15]	2011	USA	20	Retrospective case series	Post-laryngectomy pharyngocutaneous fistulas requiring free flap repair; adjunctive procedures often required for definitive closure
Iteld and Yu [16]	2007	USA	9	Retrospective case series	Anterolateral thigh free flaps for pharyngocutaneous fistula after radiotherapy and salvage laryngectomy; all reported flaps survived
Fodor et al. [17]	2019	Romania	8	Retrospective case series	Chimeric lateral arm free flap achieved reported flap survival in all patients; oral feeding restored in the cohort
Yoo et al. [18]	2006	South Korea	2	Case series	Cork-design radial forearm free flaps used to obliterate pharyngocutaneous fistulas after total laryngectomy and radiotherapy
Bao and Li [19]	2023	China	1	Case report	Radial forearm free flap combined with enteral tube strategy for refractory pharyngocutaneous fistula
Percival and Earley [20]	1989	United Kingdom	NR	Technical clinical report	Radial forearm free flap described for pharyngostome closure using external cover or double-paddle lining concepts
Tan et al. [21]	2007	Turkey	1	Case report	Free dorsalis pedis flap used after failed previous local flap management of a large pharyngocutaneous fistula
Hirano et al. [22]	2017	Japan	1	Case report	Free jejunal patch flap used for large pharyngocutaneous fistula after salvage laryngectomy
Salgado et al. [23]	2003	Taiwan/USA	9	Case series	Tissue-plug technique reported for critical oropharyngocutaneous fistulas after microsurgical head and neck reconstruction
McLean et al. [24]	2012	USA	47 PCF; 22 surgical closures	Retrospective cohort	Mixed surgical management of post-laryngectomy pharyngocutaneous fistulas; small free jejunal subgroup limited direct extraction
de Weerd et al. [25]	2007	Norway	1	Case report	Composite free latissimus dorsi-serratus flap closed a high-output enterocutaneous fistula with abdominal wall defect
Lellouch et al. [26]	2023	France/USA	1	Case report	Two-stage free latissimus dorsi flap used for voluminous enterocutaneous fistula in Ehlers-Danlos syndrome
Wong et al. [27]	2009	Taiwan/USA	Mixed abdominal wall series	Retrospective case series	Free flaps used for complex abdominal wall reconstruction; fistula outcomes not uniformly separable
Sakuraba et al. [28]	2009	Japan	1	Case report	Superior gluteal artery perforator flap reconstruction reported for enterocutaneous fistula

Patient and Fistula Characteristics

The directly relevant evidence is clearly dominated by pharyngocutaneous and pharyngostome fistulas after laryngectomy, pharyngolaryngectomy, salvage oncologic surgery, and radiotherapy, while abdominal enterocutaneous fistula reports were uncommon and generally represented exceptional salvage cases in hostile abdominal wounds, abdominal wall defects, connective tissue disorder, or complex reconstructive settings. Prior failed repair, radiotherapy, scarred local tissues, contaminated fields, and depleted recipient tissues were recurring indications for free tissue transfer [14-28]. Fistula categories and flap patterns are summarized in Table 2.

**Table 2:** Fistula type, flap type, and surgical pattern.

Fistula category	Representative studies	Free flap types reported	Interpretation
Pharyngostome/Pharyngocutaneous fistula	Llorente et al. [14]; Bohannon et al. [15]; Iteld and Yu [16]; Fodor et al. [17]; Yoo et al. [18]; Bao and Li [19]; Percival and Earley [20]; Tan et al. [21]; Hirano et al. [22]	Anterolateral thigh, radial forearm, lateral arm, jejunal patch, dorsalis pedis, septocutaneous variants	Best-supported indication; high morbidity and frequent need for adjunctive procedures remain important
Oropharyngocutaneous fistula	Salgado et al. [23]	Tissue-plug/double-layer free flap concepts	Supports tract obliteration and lining plus cover principles in irradiated or previously reconstructed head and neck fields
Enterocutaneous abdominal fistula	de Weerd et al. [25]; Lellouch et al. [26]; Wong et al. [27]; Sakuraba et al. [28]	Latissimus dorsi-serratus composite flap, latissimus dorsi flap, abdominal wall free flap/perforator approaches	Evidence limited to exceptional salvage cases; unsuitable for pooled efficacy estimation
Mixed post-laryngectomy surgical fistula cohorts	McLean et al. [24]	Mostly pedicled/local flaps with small free jejunal subgroup	Useful contextual evidence but not cleanly extractable for free flap-specific pooled outcomes

Surgical Characteristics

The reconstructive need to select the free flap included internal lining, external cover, tissue bulk to obliterate dead space, infection control, and the possibility of recipient vessels. In head and neck fistulas, it was common to use radial forearm and anterolateral thigh flaps. In selected technical situations, lateral arm, jejunal patch, dorsalis pedis, septocutaneous, latissimus dorsi, and composite flap techniques were reported. The choice of recipient vessels was inconsistently described and did not allow comparison of the vessel choice, flap design, and failure risk among studies.

Primary Outcome: Closure of Definite Fistula

Reported definitive closure in small case reports and in technical series was generally reported as successful, but such information is susceptible to publication bias. The bigger series presented a more careful view. The literature on transfer of free flaps in the pharyngocutaneous fistulas after laryngectomy was inconclusive in terms of closure; sometimes the use of additional procedures and staged management became necessary. Restoration of oral feeding in the vast majority of oral feeders who could be assessed survived with pharyngostome reconstruction in large numbers, although the cost of complication and revision was high. No pooled definitive closure rate was determined as there was insufficient comparability in definitions of closure, anatomy of fistula, adjunctive treatment, follow-up time, and denominators.

Secondary Outcomes

Explicit extractable flap survival numerators were available for 25 patients across three small series and six single-patient reports, and all 25 flaps survived. This descriptive figure must not be interpreted as an effectiveness estimate because larger cohorts reported outcomes qualitatively or in nonseparable subgroups, and successful cases are more likely to be published. Flap survival was not equivalent to clinical recovery: persistent salivary leakage, wound breakdown, stenosis, late refistulization, and further operations occurred despite a viable flap. Recurrence reporting was inconsistent. Reported complications included infection, wound dehiscence, partial or total flap necrosis, persistent leak, stenosis, donor-site morbidity, reoperation, carotid blowout in high-risk neck cases, and mortality in complex salvage populations. Primary and secondary outcomes are summarized in Table 3.

**Table 3:** Primary and secondary outcomes reported across studies.

Study	Definitive closure	Flap survival	Recurrence	Complications	Functional recovery
Llorente et al. [14]	Reported in most evaluable patients	Reported; flap necrosis in a minority	Variable reporting	High overall complication and revision burden	Oral feeding restored in most evaluable survivors
Bohannon et al. [15]	Free flap alone insufficient in some; adjunctive procedures improved final closure	High but not identical to closure	Late refistulization reported	Leak, infection, carotid blowout, and flap loss reported	Closure enabled progression toward oral intake in selected patients
Iteld and Yu [16]	Successful repair reported	All flaps survived	Not consistently extractable	Limited in abstract-level data	Short hospital stay reported
Fodor et al. [17]	Successful closure pattern reported	All flaps survived	Not prominent in abstract-level data	Small fistula/dehiscence in isolated cases	All patients resumed oral eating
Yoo et al. [18]	Successful obliteration in two cases	Successful	None reported in the abstract	No major complications reported	Functional restoration implied
Bao and Li [19]	Successful case-level closure described	Successful	Not applicable	Case-level reporting only	Enteral support used as an adjunct
Percival and Earley [20]	Technique-focused report	Not reported	Not reported	Not reported	Not reported
Tan et al. [21]	Successful case-level closure	Successful	No recurrence reported during follow-up	No major donor-site complication reported	Oral diet restored
Hirano et al. [22]	Successful case-level repair	Successful	Not extractable from abstract-level data	Case-level reporting only	Digestive continuity restored
Salgado et al. [23]	Successful management described in selected critical fistulas	Not uniformly extractable	Not reported	Case-series level reporting	Supports tract obliteration strategy
McLean et al. [24]	Mixed surgical cohort; free-flap-specific closure not cleanly separable	Small free jejunal subgroup not independently extractable	Reported at mixed-cohort level	Mixed surgical complications; technique-specific attribution limited	Functional outcome not separable for free-flap subgroup
de Weerd et al. [25]	Successful high-output enterocutaneous fistula closure	Successful	No recurrence at reported follow-up	Complex abdominal salvage setting	Oral intake restored after staged recovery
Lellouch et al. [26]	Successful reconstruction described	Successful	Case-level reporting only	High-complexity patient context	Abdominal wall restoration emphasized
Wong et al. [27]	Complex abdominal wall reconstruction series; fistula closure not uniformly separable	Free flap survival reported within reconstruction cohort	Not uniformly extractable	Complications reported at the cohort level	Abdominal wall restoration emphasized, not fistula-specific
Sakuraba et al. [28]	Successful enterocutaneous fistula reconstruction reported	Successful	Case-level reporting only	Single-case surgical morbidity reporting	Fistula reconstruction and soft-tissue restoration described

Risk-of-Bias Assessment

Overall certainty of evidence was substantially compromised by pervasive methodological limitations, including uncontrolled study designs, small sample sizes, selective outcome reporting, inconsistent endpoint definitions, incomplete follow-up, and likely publication bias. Although larger retrospective series offered greater clinical context than isolated case reports, the absence of comparator groups and marked treatment heterogeneity precluded robust inference regarding reconstructive superiority or true closure effectiveness. Risk-of-bias judgments are summarized in Table 4.

**Table 4:** Risk of bias summary.

Study	Design	Main bias concerns	Overall judgment
Llorente et al. [14]	Retrospective case series	Selection bias, heterogeneous defects, variable adjuncts	Moderate
Bohannon et al. [15]	Retrospective case series	Salvage-only population; adjunctive procedures confound closure attribution	Moderate to high
Iteld and Yu [16]	Retrospective case series	Small sample, no comparator, timing differences	High
Fodor et al. [17]	Retrospective case series	Small cohort, limited long-term recurrence data	High
Yoo et al. [18]	Case series	Two patients only; publication bias	High
Bao and Li [19]	Case report	Single case; no generalizability	High
Percival and Earley [20]	Technical clinical report	Limited extractable outcomes	High
Tan et al. [21]	Case report	Single successful case; no denominator	High
Hirano et al. [22]	Case report	Single case; limited comparative value	High
Salgado et al. [23]	Case series	Selected critical fistulas; technique-specific reporting	High
McLean et al. [24]	Retrospective cohort	Mixed techniques; free-flap-specific outcomes not cleanly separable	Moderate to high
de Weerd et al. [25]	Case report	Single extreme abdominal case	High
Lellouch et al. [26]	Case report	Single rare connective-tissue disorder case	High
Wong et al. [27]	Retrospective case series	Complex abdominal wall cohort; fistula outcomes not uniform	Moderate to high
Sakuraba et al. [28]	Case report	Single case; limited comparability	High

Summary of Results

The evidence justifies microvascular free flap transfer as a plausible salvage solution to recalcitrant digestive fistulas and pharyngocutaneous fistulas and/or pharyngostome fistulas in irradiated necks or necks that have undergone surgery. The most justifiable explanation is that vascularized free tissue has the potential to substitute for compromised tissue, offer lining and cover, obliterate dead space, and provide a better opportunity for long-term closure in selected patients. The least strong element of evidence is the quantitative certainty. The possibility of having one common rate of closure in all digestive fistulas was not justifiable, as the literature lumped together various organs, fistula biology, reconstructive objectives, and outcome measures.

Discussion

This review supports a narrow conclusion: microvascular free flap transfer is a salvage reconstructive option rather than a first-line treatment for all digestive fistulas. The most directly relevant evidence concerns post-laryngectomy pharyngocutaneous and pharyngostome defects in irradiated, infected, depleted, or repeatedly operated necks. In these fields, imported vascularized tissue can replace compromised lining and cover, obliterate dead space, and separate contaminated surfaces [6,14-26].

Flap survival cannot be equated with durable fistula closure. Larger head and neck series reported complications, revisions, and adjunctive procedures, while isolated successful cases cannot estimate true effectiveness because publication bias is likely [13-26,28]. The abdominal enterocutaneous literature is weaker and consists mainly of exceptional salvage reports [25-28]. These differences justify site-stratified interpretation and preclude a universal closure estimate.

Comparison With Existing Literature

The findings are consistent with broader reconstructive literature showing the value of vascularized tissue in hostile, irradiated, or previously operated fields [6,8,9]. The present review differs from generalized syntheses by not collapsing pharyngocutaneous, esophageal, enterocutaneous, colorectal/perineal, and pancreatic fistulas into one headline closure rate. A pharyngocutaneous fistula after salvage laryngectomy is biologically and technically different from a high-output enterocutaneous fistula in an open abdomen; recipient vessels, contamination, tissue planes, lining requirements, and functional endpoints are not interchangeable [10,11,14,15,25-28].

Clinical and Technical Implications

Microvascular reconstruction should be considered when local repair is biologically unlikely to succeed, particularly in irradiated or surgically exhausted head and neck fields requiring lining, cover, or both. Thin fasciocutaneous flaps may provide pliable lining and external cover; jejunal patches can restore mucosal continuity; chimeric or composite flaps can combine lining, bulk, and skin; and larger muscle or perforator-based flaps may be required for abdominal dead-space and wall restoration. Recipient-vessel planning is central in irradiated and reoperative fields [14-28]. Current abdominal evidence is too sparse to support routine use and should be limited to individualized salvage in specialist centers.

Limitations of the Evidence

Most studies were uncontrolled and retrospective, and many were single case reports, creating serious selection and reporting bias and likely overestimating success [13-23,25,26,28]. Outcome definitions varied, flap survival was often reported more clearly than definitive closure, follow-up was inconsistent, recurrence was likely underreported, and complication grading was not standardized. Mixed cohorts included techniques or indications that could not be separated, preventing strong comparative inferences [24,27].

Additional limitations were the English-language restriction, possible database retrieval bias, lack of systematic grey literature searching, inclusion of conference abstracts only when found through supplementary searching, inability to formally assess publication bias, absence of a preserved day-level search log, and lack of documented duplicate screening and extraction.

Strengths of the Review

The review distinguishes directly eligible studies from contextual reports, separates flap survival from fistula closure, avoids unsupported pooled estimates, and emphasizes anatomical differences rather than forcing clinically incompatible studies into a single statistic [12,13]. This conservative approach is more defensible for sparse, heterogeneous surgical evidence.

Future Research Directions

Future studies should use prospective registries, standardized definitions of durable closure, minimum follow-up periods, explicit recurrence reporting, complication grading, and patient-centered functional endpoints [12,13]. Comparative cohorts should distinguish prophylactic reconstruction from treatment of established fistulas and report free-flap, pedicled-flap, local-flap, and nonoperative adjunct outcomes independently. Recipient-vessel strategy, radiation exposure, nutritional status, fistula output, infection control, and timing of reconstruction should be standardized [14,15,24-28].

## Conclusions

Microvascular free flap transfer is a salvage option for selected recalcitrant digestive fistulas, particularly pharyngocutaneous and pharyngostome fistulas in irradiated or surgically depleted head and neck fields. It provides vascularized lining or cover, dead-space obliteration, and replacement of damaged tissue. The evidence is very low in certainty and is dominated by retrospective series and case reports; therefore, the descriptive 25/25 flap survival figure from small extractable reports must not be generalized, and a single pooled closure rate across digestive fistula sites is not justified. Future studies require standardized definitions, prospective data collection, longer follow-up, and anatomically stratified reporting.
